# Research on land cover type classification method based on improved MaskFormer for remote sensing images

**DOI:** 10.7717/peerj-cs.1222

**Published:** 2023-02-03

**Authors:** Haiwen Chen, Lu Wang, Lei Zhang, Yanping Li, Zhongrong Xu, Lulu Cui, Xilai Li

**Affiliations:** 1 Department of Computer Technology and Application, Qinghai University, Xining, Qinghai, China; 2 College of Computer Science, Sichuan University, Chengdu, Sichuan, China; 3 College of Agricultrue and Animal Husbandry, Qinghai University, Xining, Qinghai, China

**Keywords:** Remote-sensing image, Land cover type classification, Neural network, Transformer, Vision transformer

## Abstract

High-resolution remote sensing images have the characteristics of wide imaging coverage, rich spectral information and unobstructed by terrain and features. All of them provide convenient conditions for people to study land cover types. However, most existing remote sensing image land cover datasets are only labeled with some remote sensing images of low elevation plain areas, which is highly different from the topography and landscape of highland mountainous areas. In this study, we construct a Qilian County grassland ecological element dataset to provide data support for highland ecological protection. To highlight the characteristics of vegetation, our dataset only includes the RGB spectrum fused with the near-infrared spectrum. We then propose a segmentation network, namely, the Shunted-MaskFormer network, by using a mask-based classification method, a multi-scale, high-efficiency feature extraction module and a data-dependent upsampling method. The extraction of grassland land types from 2 m resolution remote sensing images in Qilian County was completed, and the generalization ability of the model on a small Gaofen Image Dataset (GID) verified. Results: (1) The MIoU of the optimised network model in the Qilian grassland dataset reached 80.75%, which is 2.37% higher compared to the suboptimal results; (2) the optimized network model achieves better segmentation results even for small sample classes in data sets with unbalanced sample distribution; (3) the highest MIOU of 72.3% is achieved in the GID dataset of open remote sensing images containing five categories; (4) the size of the optimized model is only one-third of the sub-optimal model.

## Introduction

With the development of remote sensing technology, the resolution of remote sensing images is constantly improved. Satellite images are widely used in the research of land cover type classification methods in large areas, especially in urban planning (Zhang et al., 2018), ecological environment monitoring (Treitz, 2000), ecological value estimation (Sutton & Costanza, 2002) and other fields. At present, the interpretation of satellite images information mainly relies on visual interpretation, machine interpretation and deep learning methods. Visual interpretation has the highest accuracy, but the time and labor cost of manual interpretation of satellite images is often greater than its practical value. Machine interpretation method utilizes the characteristics of satellite images with multiple bands. The use of machine learning methods to interpret remote sensing images has certain application value, such as satellite images classification based on the support vector machine (SVM) algorithm (Li, Lu & Chen, 2015), and remote sensing image forest vegetation classification based on the random forest model (Juel et al., 2015). However, the spectral information has the problems of “same thing different spectrum” and “foreign matter same spectrum”, which lead to poor segmentation accuracy and inaccurate boundary segmentation. These algorithms usually handle only few categories and cannot cope with complex scenes due to the limitations of artificial features.

Since the AlexNet model (Krizhevsky, Sutskever & Hinton, 2017) won the champion in the 2012 ImageNet Competition, deep learning networks have been widely used in the field of computer vision. The proposal of fully convolutional networks (FCN) (Long, Shelhamer & Darrell, 2015) provides a solution for the pixel-by-pixel classification tasks of images. However, FCN does not effectively utilize shallow features leading to too coarse segmentation results. In order to effectively fuse features at different levels, U-Net (Ronneberger, Fischer & Brox, 2015) and SegNet (Badrinarayanan, Kendall & Cipolla, 2017) use encoder–decoder structure and skip connection structure to enrich feature map. Pan et al. (2020) studied segmentation and classification for urban village using a worldview satellite image based on the U-Net model and (Weng et al., 2020) realized water areas segmentation from remote sensing images using a separable residual SegNet network. DeepLab series models (Chen et al., 2014; Chen et al., 2017; Chen et al., 2018a; Chen et al., 2018b), PSPNet (Zhao et al., 2017) network uses feature pyramids and atrous convolutions to improve the feature fusion capability of the network. Lin et al. (2020) studied road extraction from very-high-resolution remote sensing images *via* a nested SE-Deeplab model, and Yuan, Wang & Xu (2022) researched the extraction of building from remote sensing images based on shift pooling PSPNet. Naushad, Kaur & Ghaderpour (2021) completed the land use and land cover classification of sensing images based on transfer learning. Segmentation methods based on convolutional neural network (CNN) (Lecun et al., 1998) completes the extraction of features by concatenating a series of convolution and pooling operations such as VggNet (Simonyan & Zisserman, 2014), ResNet (He et al., 2016) and HRNet (Sun et al., 2019). In this process, because of the limited size of the convolutional kernel, the network only captures the local feature information of the image, but it lacks an understanding of the global information of the image. In addition, the convolutional neural network is sensitive to the rotation angle of the image. Different rotation angles of the same image will activate different neurons. Although this problem can be alleviated by data augmentation, it also increases the difficulty of training the network. Therefore, some scholars have applied the transformer (Vaswani et al., 2017) model from the field of natural language processing to the field of computer vision. Based on the self-attention mechanism, Vision Transformer can model the global information of the image, mining the long-distance relationship and parallel calculation, which has achieved a good effect in the field of computer vision.

Dosovitskiy et al. (2020) proposed the Vision Transformer network in 2020. The author divided the image into many sub-blocks and composed these sub-blocks into linear embedding sequences to simulate phrase sequence input in natural language processing. Vision Transformer provides a new model for the application of transformer in the field of computer vision and achieves competitive results in ImageNet (Deng et al., 2009) dataset. Since 2020, Transformer-based vision models have developed rapidly. Wang et al. (2021) introduced a pyramid structure to propose the Pyramid Vision Transformer (PVT) for dense prediction tasks. Liu et al. (2021) proposed the Swin Transformer with sliding window and hierarchical design. Ren et al. (2022) proposed the Shunted Transformer that mixes features at multiple scales, allowing different attention heads within the same layer to model objects at various scales simultaneously. Many scholars applied the transformer-based visual model to the interpretation of remote sensing images. For example, Xu et al. (2021b) used Swin Transformer as a feature extraction network to complete the remote sensing image segmentation task, and Xu et al. (2021a) used Swin Trasformer based on remote sensing images for target detection and instance segmentation.

Although the above works are based on different methods to complete the segmentation task, most of them consider the semantic segmentation task and the instance segmentation task as two different paradigms. Among them, semantic segmentation and instance segmentation are regarded as per-pixel classification task and mask classification task respectively. Cheng, Schwing & Kirillov (2021) proposed that MaskFormer unified the semantic segmentation task and the instance segmentation task using the mask classification paradigm, which outperformed the current pixel-by-pixel segmentation processing paradigm in performance. In addition, most of the current studies are based on ISPRS Vaihingen (ISPRS, 2022a), ISPRS Potsdam (ISPRS, 2022b) and other datasets to segment land cover types detection methods in urban scenes. Compared with buildings and roads in urban scenes with relatively regular shapes, the extraction of irregular land cover types for field grasslands is more complex.

In our work, we address the above issues and improve existing methods. We make the following contributions in this article:

(1) We provide support for the extraction of wild grassland land cover types in high-altitude mountain areas, and produced a dataset of grassland land cover types in Qilian County.
(2) We propose a Shunted-MaskFormer network for the classification of land cover types from high-resolution satellite images. Our network offers better results on a smaller scale compared with other advanced networks.
(3) We use the mask classification approach for image segmentation tasks to obtain higher segmentation accuracy while effectively suppressing the influence of data imbalance in satellite image datasets.

### Dataset construction

#### Overview of the study area

**Figure 1:**
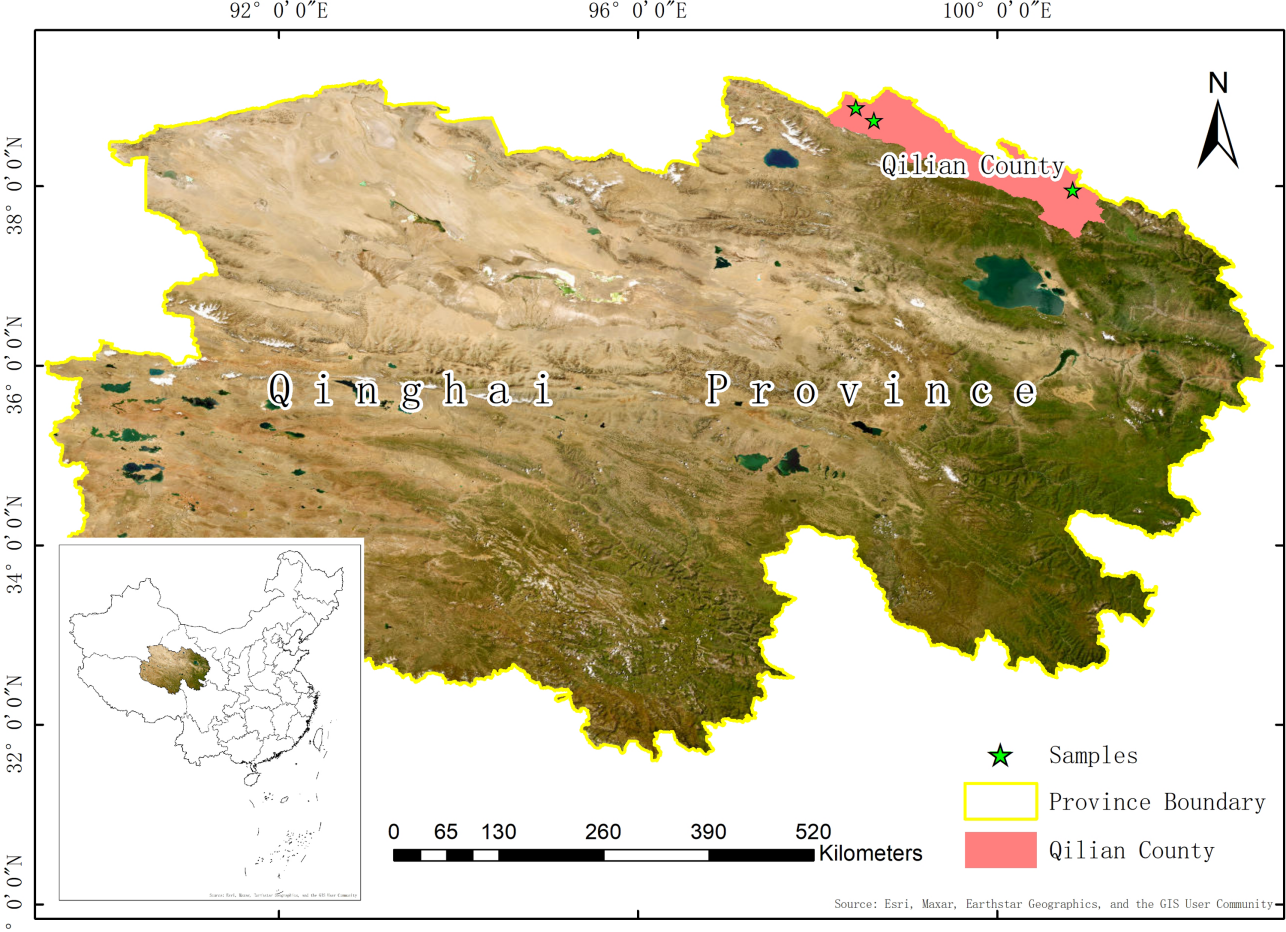
Qilian county study area, Qinghai province.

The study area, Qilian County, is located in the northern part of Qinghai Province, China. The county is located in the Qilian Mountains with an area of approximately 13,900 km^2^ and an average altitude of 2,787 m. The types of land used in Qilian County are complex and diverse, mainly consisting of grassland, bare land and woodland. The grasslands are mainly natural pasture and other grasslands, and there are relatively few other land types such as transport and building land. Figure 1 shows our study area.

#### Dataset production

**Figure 2:**
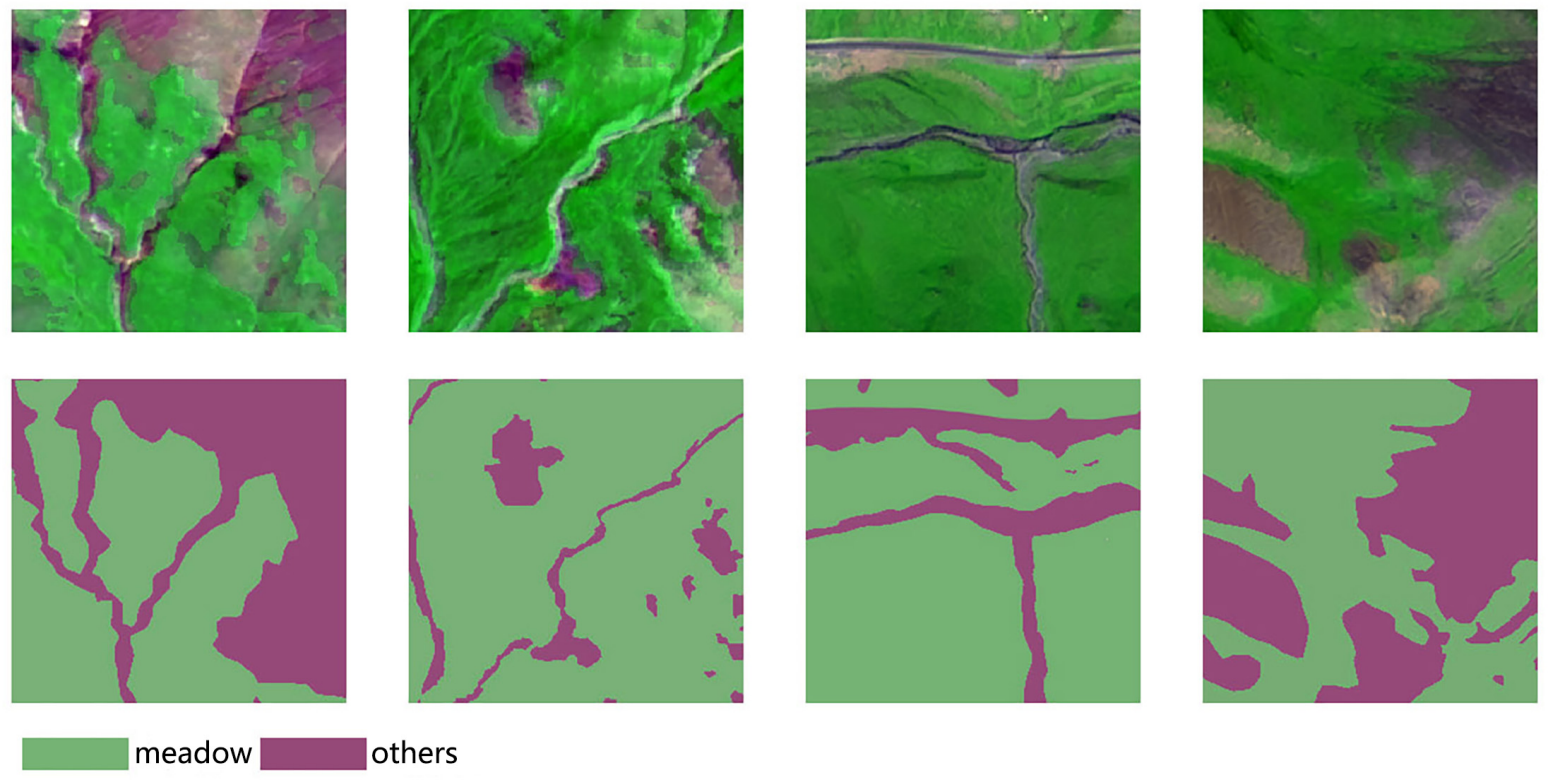
Several images from Qilian grassland dataset.

In this study, the remote sensing images of the Qilian County area were acquired between June and October 2020. A total of 24 scenes are from Gaofen-1, Gaofen-6 and Ziyuan-3 satellites. These original satellite images were from the Natural Resources Remote Sensing Center of Qinghai Province. The original image is a multispectral image with four bands: red, green, blue, and near-infrared. We enhance the vegetation in the image in order to differentiate vegetation from other land covers. Specifically, we remove the near-infrared band, multiply the near-infrared value by 0.2 and add it to the green band. The images with less cloud interference are selected from the original images and then processed by orthorectification, image fusion and image cropping to obtain high-resolution images with a spatial resolution of 2 m in the study area (Li et al., 2022). Qilian County is located at a high altitude and highland hills and gentle slopes dominate the terrain. In this study, several locations were randomly selected in the area containing the above two types of landforms to annotate and create a grassland dataset. The images are annotated as grassland and other categories using ArcGIS tools, and saved as shape-format data. We crop both the label image and the satellite image with base size 256 × 256 pixels, resulting in a total of 820 images. The grassland dataset of Qilian County is randomly divided into training set (80%, 656 images), the validation set (10%, 82 images) and the test set (10%, 82 images). Some of the dataset images and corresponding labels are shown in Fig. 2.

## Materials & Methods

In this study, the Shunted-MaskFormer network is improved based on the MaskFormer network. We employ a more efficient feature extraction network and a data-dependent decoder to restore the feature map to its original size.

### Overall model framework

**Figure 3:**
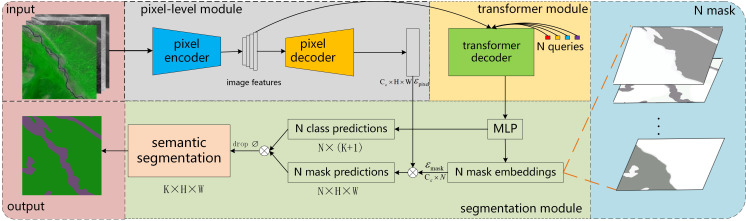
Architecture of MaskFormer. We use a pixel encoder to extract image features. A pixel decoder upsampling the feature map to obtain the *ɛ*_pixel_. A transformer decoder uses image features to produce N mask embeddings. Finally, in the semantic segmentation module, we use matrix multiplication for N class predictions and N mask predictions to obtain the final prediction results.

The MaskFormer network structure (Cheng, Schwing & Kirillov, 2021) treats the semantic segmentation task of pixel-by-pixel classification as a mask classification task, which predicts a set of binary masks, and each mask is associated with a global category label to complete the image segmentation task, and the overall framework is shown in Fig. 3.

The model framework consists of three parts: the pixel-level module, the transformer module, and segmentation module, which complete the feature extraction and feature map upsampling functions, the mask prediction function, and the final segmentation function, respectively. Specifically, the pixel-level module uses an encoder–decoder structure, with the input being an image of *C* × *H* × *W* and the output being *ɛ*_*pixel*_ ∈ ℝ^*C*_*ɛ*_×*N*×*W*^; the transformer module uses the standard decoder structure to take as input the feature map output from the encoder in the pixel-level module and the positional embedding of the N learnable positions, and the output is Q ∈ ℝ^*C*_*Q*_×*N*^; the segmentation module uses Q output from the transformer module to obtain the category probability ${ \left\{ {p}_{i}\in {R}^{K+1} \right\} }_{i=1}^{N}$ for each segment using a linear classifier, followed by a softmax activation. And transform Q through the multi-layer perceptron of the two hidden layers into *ɛ*_*mask*_ ∈ ℝ^*C*_*ɛ*_×*N*^. Then the N predicted binary masks are generated by dot product between the pixel embedding *ɛ*_*pixel*_ and the mask embedding *ɛ*_*mask*_. Finally, in the segmentation module, we use simple matrix multiplication to get the final prediction matrix *K* × *H* × *W* after removing the empty target category. In this study, we use a more practical feature extraction network and upsampling method based on the MaskFormer network to reduce network complexity and improve the accuracy of image segmentation.

### Encoders

**Figure 4:**
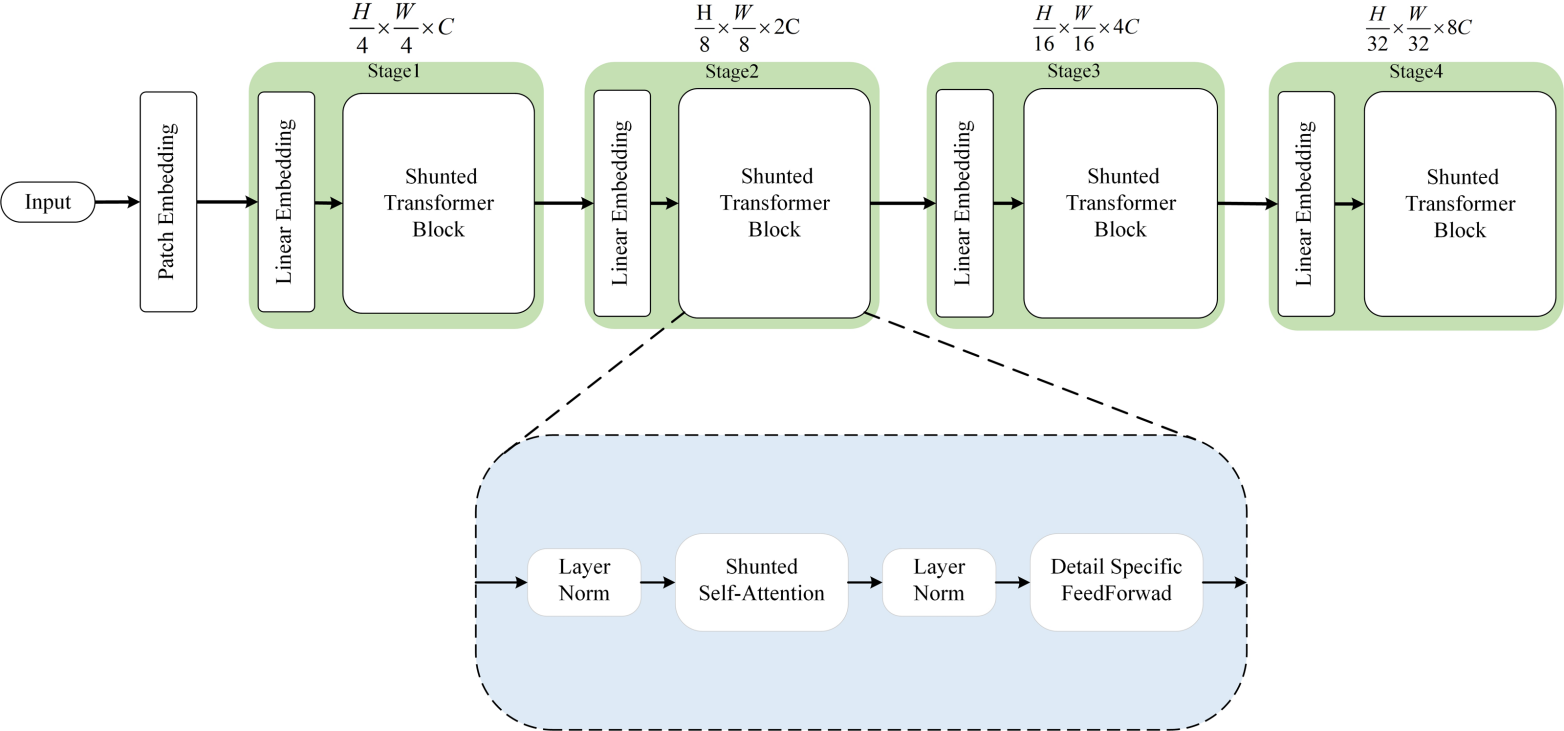
The overall architecture of the Shunted Transformer and details of the Shunted Self-Attention block.

We use the Shunted Transformer (Ren et al., 2022) as the feature extraction network, and the network structure is shown in Fig. 4. The whole framework consists of a Patch Embedding module and four cascade modules to produce four resolution outputs. Each module contains a linear embedding and a Shunted Transformer Block module. The Shunted Transformer Block module contains two normalisation layers, Shunted self-attention and Detail Specific FeedForwad. The LN layer normalises the data to make the training process more stable, while the Shunted self-attention layer captures information at different granularities for each attention head, reducing computational effort while fusing multi-scale attention information. Compared with the traditional feedforward layer, the Detail Specific FeedForwad layer adds a detail convolution branch to specify the details in the feedforward layer to supplement feature information. The details are as follows: Given an input of 3 × *H* × *W* (3 for RGB channels), the patch embedding module first generates a non-overlapping input sequence of size $ \frac{H}{4} \times \frac{W}{4} $ using the convolutional layers of 7 × 7 and 3 × 3.

At this point, the resolution of the original input *H* × *W* is reduced to $ \frac{H}{4} \times \frac{W}{4} $ and the dimensionality is changed from 3 to 48. The linear embedding layer then maps the dimensionality from 48 to C and feeds the features into the Shunted Transformer module. Each attention head in the module computes attention at different scales and captures information of different granularities for global modelling. In each subsequent iteration module, the resolution of the feature map is reduced to half of the output of the previous module, and the number of channels is doubled.

### Decoders

**Figure 5:**
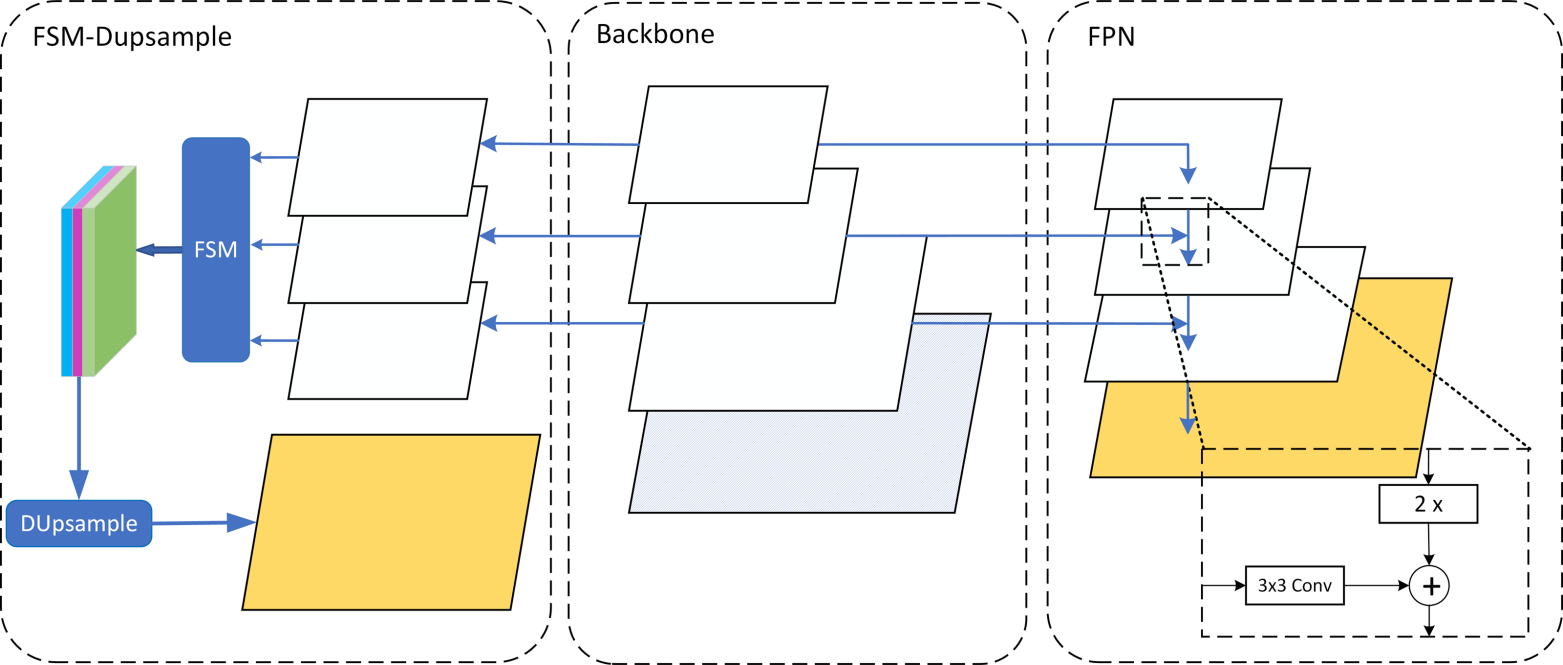
Upsampling method. Middle: backbone denotes the feature extraction process from shallow to deep. Left: the structure of the FSM-DUpsample method. Right: the structure of the FPN method.

In the decoder part, the original model adopts the feature pyramid network (FPN) (Lin et al., 2017) structure, using bilinear interpolation to upsample the feature map twice, and then fuses the feature map layer by layer from deep to shallow to the original image size. However, the relatively coarse use of nearest neighbor interpolation to upsample feature maps, whose unlearnability may lead to ineffective transfer of high-level feature information. The decoder of the Shunted-MaskFormer network adopts a data-dependent upsampling method. First, the feature maps of different resolutions are uniformly downsampled to the same resolution as the deepest feature map. Second, the feature selection module is used to adaptively recalibrate channel-wise feature response. Finally use the DUpsample (Tian et al., 2019) module to restore the feature map to the original resolution. The entire upsampling process is shown in Fig. 5.

### Feature selection module

Compared with the simple use the convolution of 3 × 3 in FPN, before using the feature map extracted by the encoder, it is important to emphasize the feature maps that contain a lot of spatial detail information, while suppressing redundant feature maps. The Squeeze and Excitation (SE) module is proposed by Hu, Shen & Sun (2018). This structure takes into account the different importance of each channel to different categories and obtains the weight vectors of different channels mainly through two operations, squeeze and excitation. The details are as follows, the squeeze operation is used for the down-sampled feature map to extract the global information of different channels using global average pooling, and the excitation operation is used to calculate the dependencies between different channels and output the weight vector. Next, the original feature map is scaled using the weight vectors and added to the original feature map to form a residual structure. The residual structure is used to avoid some channels being over-scaled or suppressed, and the process is defined as:

(1) \begin{eqnarray*}y& =x\ast u+x\end{eqnarray*}

(2) \begin{eqnarray*}u& ={f}_{E} \left( z \right) ,\end{eqnarray*}

(3) \begin{eqnarray*}z& ={f}_{S} \left( x \right) \end{eqnarray*}

where: *x* is the feature map input to the feature extraction module; ${f}_{S} \left( \cdot \right) $ is the squeeze operation; *z* is the global information for each channel; ${f}_{E} \left( \cdot \right) $ is the excitation operation; *u* is the calculated weight vector; *y* is the output of the feature selection module.

### DUpsample module

The final layer of the decoder is usually a bilinear upsampling process that restores the feature map to its original resolution. This upsampling method is data-independent and does not consider the correlation between each pixel. Such an upsampling process may lead to suboptimal results. Tian et al. (2019) proposed a data-dependent upsampling method (DUpsample) to replace the bilinear interpolation method. DUpsample exploits the spatial redundancy in segmentation labels to accurately restore the feature map to the original scale, and does not require multiple upsampling strides, thus reducing the framework’s computation time and memory footprint.

In the training process, we no longer use the interpolation method to upsample the feature map, but complete the upsampling process of the feature map *F* by finding the reconstruction matrix *W*. The segmentation label *Y* is not independent and identically distributed, it contains structural information and can be compressed without causing too much loss. In order to minimize the reconstruction error, we use the linear projection method to compress *Y* to $\tilde {Y}\in {\mathbb{R}}^{\tilde {H}\times \tilde {W}\times \tilde {C}}$ with the same size as *F*. First, *Y* isdivided into sub-windows of size *r* × *r*(*r* represents the ratio of the original scale *H* to the compressed scale $\tilde {H}$), after which each sub-window is deformed into a vector *v* and multiplied by the matrix *P* to obtain *x*. Finally, stack *x* vertically and horizontally to obtain $\tilde {Y}$. The formula is expressed as:

(4) \begin{eqnarray*}x=Pv;\tilde {v}=Wx.\end{eqnarray*}

Here $P\in {\mathbb{R}}^{\tilde {C}\times N}$ is used to compress *v* to *x*, *W* is the reconstruction matrix used to reconstruct *x* back to *v*, and $\tilde {\nu }$ represents the reconstructed *v*.*W* can be found by minimizing the error between v and $\tilde {\nu }$. Use the reconstruction matrix *W* to complete the upsampling of *F* and calculate the error with *Y* as a loss function.

(5) \begin{eqnarray*}L \left( F,Y \right) =Loss \left( softmax \left( DUpsample \left( F \right) \right) ,Y \right) .\end{eqnarray*}

**Figure 6:**
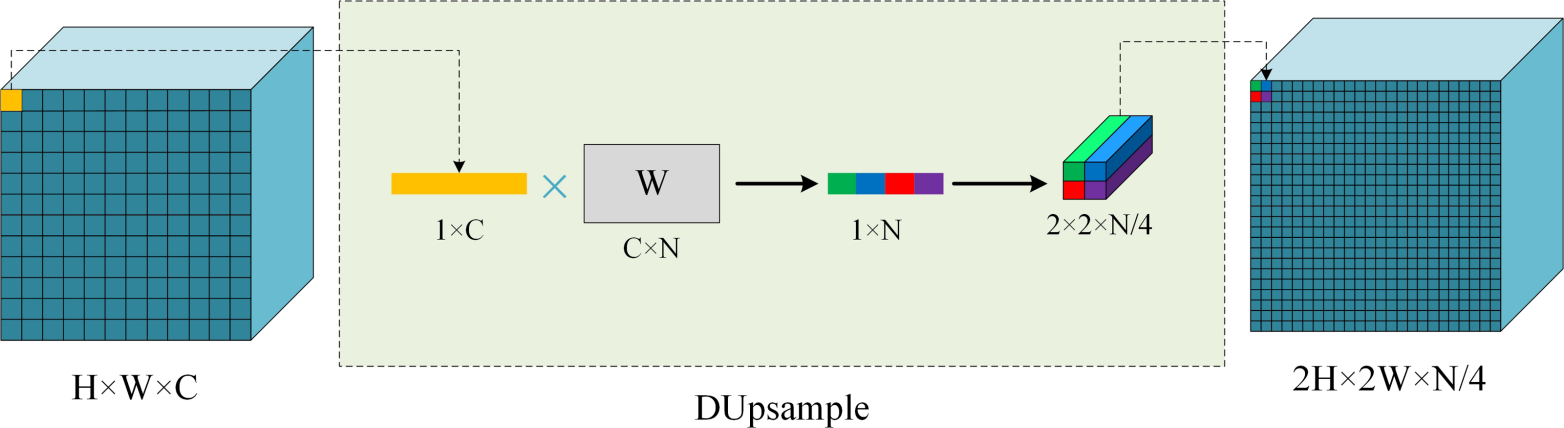
The framework with Dupsample. DUpsampling: upsample the backbone outputs feature maps twice. Left: denotes the feature maps extracted by the backbone. Right: denotes the resulting maps. W: the inverse projection matrix. In our experiment, the upsampling ratio is 8.

With linear reconstruction, DUpsample (*F*) applies a linear upsampling to each feature in the tensor *F*. This upsampling process is essentially the same as applying a 1 × 1 convolution along the spatial dimension, with the convolution kernel stored in *W*. Decompression is shown in Fig. 6.

### Experiments and analysis

#### Software and hardware

As experimental hardware, we used two GeForce GTX 1080 Ti graphics cards with a memory capacity of 11 GB each. We implemented the machine learning platform with PyTorch 1.8.1, Python 3.8 and CUDA version 10.2.

#### Training settings

##### Optimizer.

We use MMSegmentation (Contributors, 2020) and follow the default training settings for each model. More specifically, we use AdamW (Loshchilov & Hutter, 2017) and the WarmupPolyLR learning rate schedule with an initial learning rate of 10^−3^ and a weight decay of 5⋅10^−4^for ResNet (He et al., 2016) backbones, and an initial learning rate of 6⋅10^−5^ and a weight decay of 10^−2^ for Swin Transformer (Liu et al., 2021) and Shunted Transformer (Ren et al., 2022) backbones.

##### Batchsize.

We set different batch sizes for other models in the two datasets to fully use hardware resources. During the training, we set the batch size to 20 for all models except Upernet (Xiao et al., 2018) and Shunted Transformer. According to the model size, the batch size of the Swin Transformer is set to 8, and the Shunted Transformer is set to 32.

##### Pre-training.

Backbones are pre-trained on ImageNet-1K (Russakovsky et al., 2015) if not stated otherwise. U-net (Ronneberger, Fischer & Brox, 2015), SegNet (Badrinarayanan, Kendall & Cipolla, 2017), Deeplab v3+ (Chen et al., 2018a), PsPnet (Zhao et al., 2017) and Ocrnet (Yuan, Chen & Wang, 2020) used pre-trained ResNet50 (He et al., 2016) as their backbone network. Upernet (Xiao et al., 2018) used the pre-trained Swin Transformer and our method used the pre-trained Shunted Transformer.

#### Data augmentation

The inputs to the training were of three types: the original image, horizontal and vertical flip input image, and rotate the input image at any angle. All semantic segmentation networks randomly choose one or more as inputs during training to increase the diversity of the dataset.

##### Evaluation metrics.

The number of floating-point operations per second (FlOPs) and the number of model parameters (Params) are used as the model complexity metrics, and the mean intersection over union (MIoU) and mean pixel accuracy (MPA) are used as the comprehensive evaluation metrics for the accuracy of the segmentation results.

(6) \begin{eqnarray*}MIoU& = \frac{1}{k+1} \sum _{i=0}^{k} \frac{{p}_{ii}}{\sum _{j=0}^{k}{p}_{ij}+\sum _{j=0}^{k}{p}_{ji}-{p}_{ii}} \end{eqnarray*}

(7) \begin{eqnarray*}MPA& = \frac{1}{k+1} \sum _{i=0}^{k} \frac{{p}_{ii}}{\sum _{j=0}^{k}{p}_{ij}} \end{eqnarray*}

where there are *k* + 1 classes (including a background class), *p*_*ij*_ denotes the number of pixels that belong to class *i* but are predicted as class *j*, *P*_*ii*_ denotes the number of correct predictions for class *i*, *p*_*ij*_ and *p*_*ji*_ are false positive and false negative, respectively.

#### Dataset

**Figure 7:**
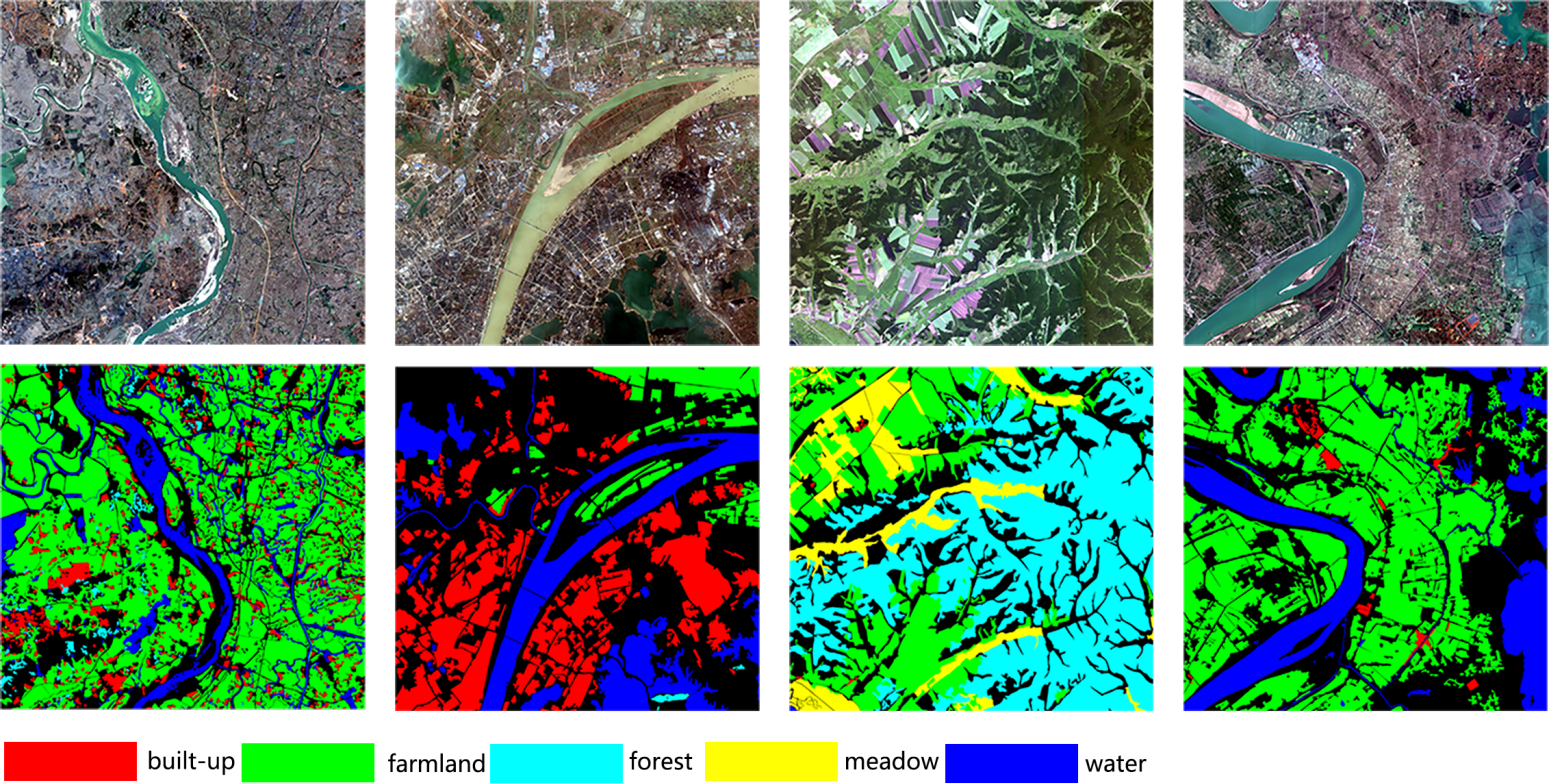
Several images from the GID dataset.

Two datasets are chosen for the experiment, the Qilian grassland dataset and the Gaofen Image Dataset (GID) (Tong et al., 2020). The construction of the Qilian grassland dataset has been discussed previously. The GID dataset contains 150 images from the Gaofen-2 satellite, each with a size of 7,200 × 6,800 pixels, containing five categories: buildings, farmland, forest, grassland and water. The original data set provides RGB images and near-infrared images. In this study only selected the red–green–blue version of the GID dataset. We choose the typical morphology of each category in the GID dataset as shown in Fig. 7.

**Figure 8:**
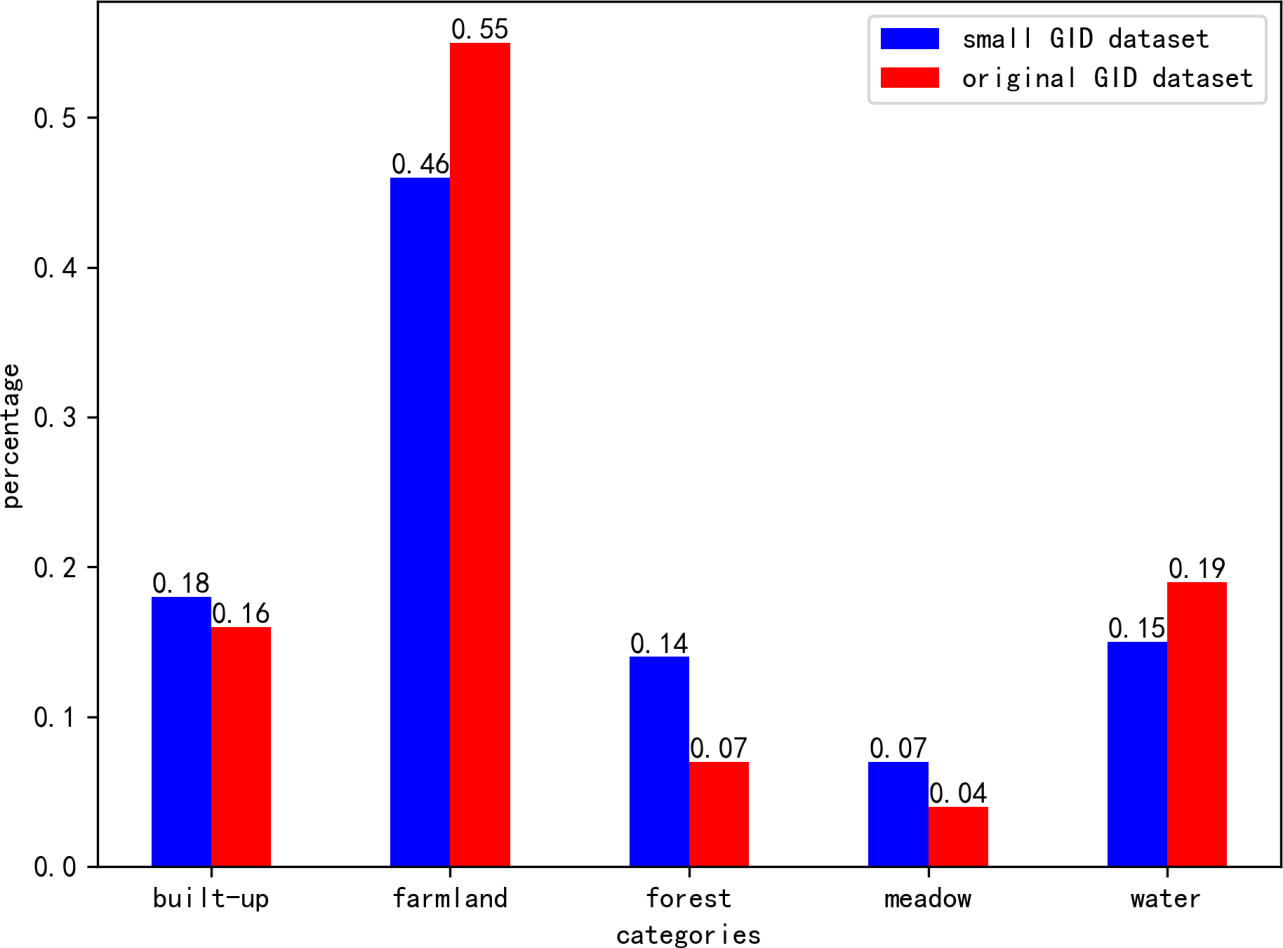
The per category distribution in each dataset. In the small GID dataset, we reduced the proportion of farmland category and water category, and increased the proportion of the other three categories.

This large dataset has an unbalanced number of samples in each category. In order to balance the number of samples in each category and accommodate the limited computational resources. We randomly selected 15 images and cropped them to a size of 256 × 256. Subsequently, 3,000 images were selected from the cropped images to constitute a small GID dataset for the experiment. The small GID dataset is randomly divided into training set (80%, 2,400 images), the validation set (10%, 300 images) and the test set (10%, 300 images). The percentage of data in each sample category is shown in Fig. 8.

## Results

### Qilian grassland dataset

We compared Shunted-Transformer with several other state-of-the-art computer vision networks on the Qilian grassland dataset for land cover types segmentation. Table 1 shows the results. DeepLab v3+ achieves the best segmentation results among several models based on convolutional neural networks, but there is still a relatively large gap in segmentation accuracy compared to the Vision Transformer network-based models. Shunted-MaskFormer achieves the highest segmentation accuracy. The MIoU and MPA of our method on this dataset are 80.75 and 88.89%. Furthermore, regarding the single-class segmentation results, the grass achieved the best result of 85.74% and the other category also achieved the best segmentation result of 75.76%.

**Table 1:** Evaluation table for land cover type segmentation in the Qilian grassland dataset.

**Methods**	**backbone**	**MIoU**	**MPA**	**Type of land cover**	
				Others	Meadow
U-net	Res50	71.79	84.80	64.63	78.95
Segnet	Res50	71.84	84.95	64.35	79.34
PsPnet	Res50	74.35	85.11	68.27	80.43
DeepLab v3+	Res50	76.33	85.20	69.41	83.24
Ocrnet	Res50	75.93	85.23	69.23	82.64
Upernet	Swin_base	78.38	86.88	72.37	84.40
Our method	Shunted_base	**80.75**	**88.89**	**75.76**	**85.74**

**Notes.**

Bold values indicate the highest values of every column.

**Figure 9:**
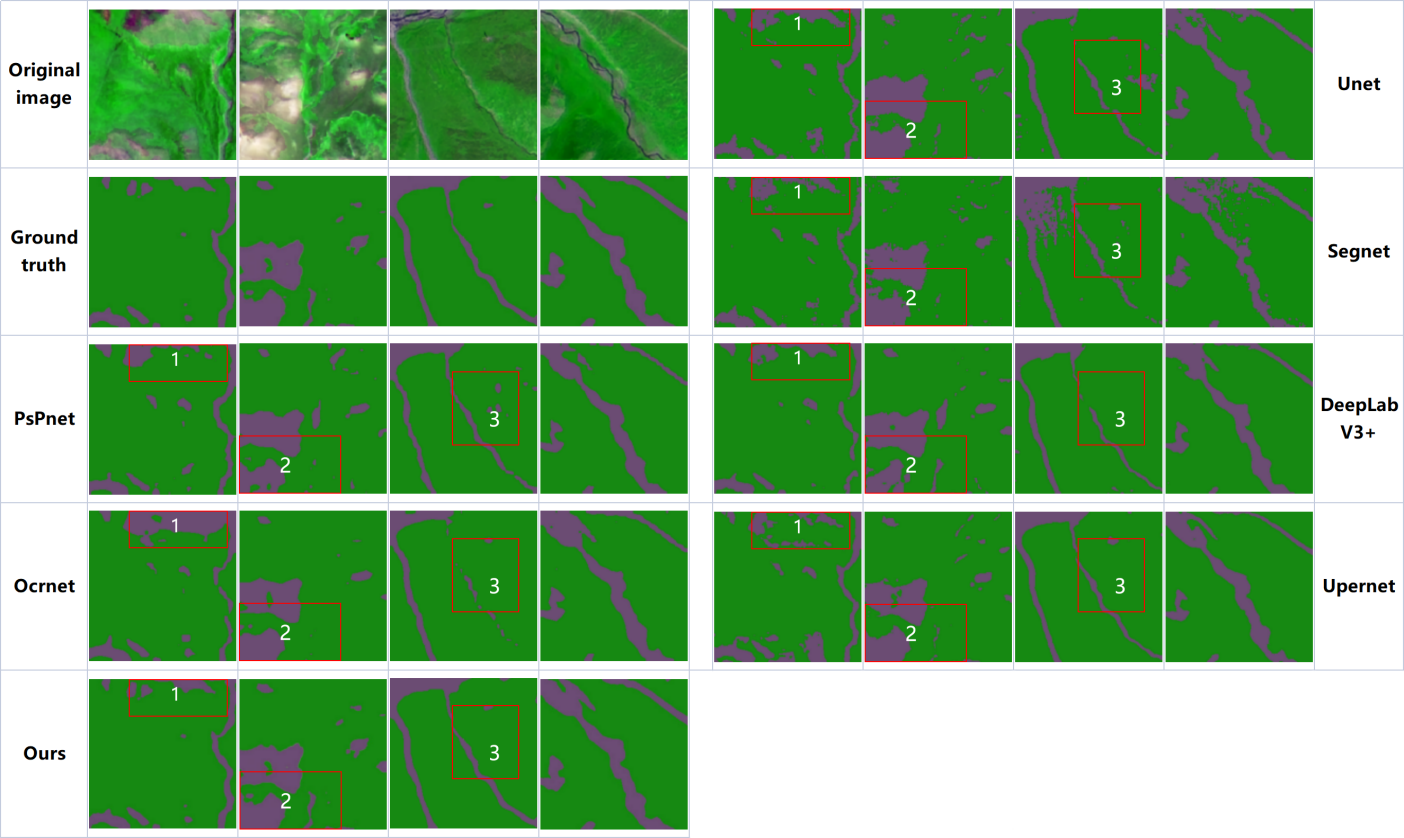
Visual comparison of the classification results of different methods on the Qilian grassland dataset. In the figure, green color represents the segmentation result of grass class and purple color represents the other class.

Figure 9 illustrates the visualization of the results of our method compared with the comparison method on the Qilian grassland dataset. From region 1 and region 2 in the figure, it can be seen that in the comparison method of a convolutional neural network, due to the inevitable spatial smooth processing of convolution kernel in the process of convolution, some small areas of other categories and grassland cannot be clearly distinguished and the edge segmentation is not satisfactory enough. Through the attention mechanism, the two segmentation networks based on Vision Transformer are modeled globally, and the segmentation results are better than convolutional neural networks overall, but Upernet overcomes the disadvantage of too smooth boundary segmentation while also leading to more fragmented meadows misclassified into other categories such as region 1. And because only at a single scale calculating attention, it leads to discontinuous boundary segmentation of grassland formed by some small rivers such as region 2 and region 3. Our method used a multi-scale feature extraction network and a data-dependent upsampling process to obtain the finest boundary segmentation results while ensuring the integrity of objects with different land types.

#### GID dataset

Table 2 shows the results of land cover types segmentation on the small GID dataset for our method and the comparison method. It can be seen that our method achieves the optimal segmentation results in the dataset. However, the improvement in MIoU accuracy is not significant, and our main advantage is that we achieve the most balanced segmentation results in each class of the dataset. From Fig. 8 we know that grass is a few-shot category in this dataset, but our method still achieves the highest segmentation accuracy. It can be seen that our method is able to overcome the effect of sample imbalance in the dataset.

**Table 2:** Evaluation table for land cover type segmentation of the GID dataset.

**Methods**	**backbone**	**MIoU**	**Type of land cover**				
			Built-Up	Farmland	Forest	Meadow	Water
U-net	Res50	64.12	65.35	62.27	60.41	50.00	82.58
Segnet	Res50	68.90	66.95	70.84	70.27	49.87	86.59
PsPnet	Res50	69.05	45.11	74.31	75.61	57.17	88.72
DeepLab v3+	Res50	69.17	44.49	76.6	76.36	56.06	88.93
Ocrnet	Res50	68.84	43.92	74.27	74.97	57.32	89.07
Upernet	Swin_base	70.96	49.54	**79.73**	**76.43**	54.35	**89.94**
Our method	Shunted_base	**72.30**	**69.87**	74.66	72.95	**58.26**	86.49

**Notes.**

Bold values indicate the highest values of every column.

### Model size analysis

Through the accuracy analysis of the above two datasets, we proved the validity and generalization ability of the model. In this section, we discuss the scale of the model. We used the number of model parameters and the number of FlOPs as the indicators of model size and complexity respectively. The image size of the input network is uniformly 256 × 256, and the number of parameters and FlOPs measured for each model are shown in Table 3. SegNet has the lowest number of parameters and FlOPs. Upernet has the largest model size with 104M parameters. Our model not only achieves the highest accuracy but also has a relatively small model scale and small complexity among all the models. Our approach achieves a good balance between segmentation accuracy and model complexity.

**Table 3:** Model scale evaluation table.

**Methods**	**backbone**	**Params(M)**	**FlOPs(G)**
U-net	Res50	16.37	23.34
Segnet	Res50	**14.86**	17.48
PsPnet	Res50	48.96	44.72
DeepLab v3+	Res50	43.58	44.05
Ocrnet	Res50	36.51	38.22
Upernet	Swin_base	104.45	31.74
Our method	Shunted_base	35.31	**10.14**

**Notes.**

Bold values indicate the best values of every column.

## Discussion

Remote sensing images are the true reflection of various types of land use on the ground, so the proportion of different land types in remote sensing image datasets is relatively unbalanced. For example, the distribution of various categories of buildings, cultivated land, and grassland in remote sensing image data in every region is usually different. The feature puts forward higher requirements for the segmentation ability of remote sensing image semantic segmentation network. The experiment results show that our method achieves high classification accuracy in small sample categories in multi-category GID datasets. It may be related to the classification strategy we adopt, which uses N categories in the Transformer module to classify (N is much greater than the actual number of categories k), and further N categories are mapped to the actual category k in the Segmentation module. Therefore, the impact of data set sample imbalance has been reduced.

Due to our network has smaller scale and lower computing requirements, our method can obtain competitive results compared with other advanced image segmentation methods. Under the condition of limited computing resources, the relatively larger batch size can be supported in our method. However, experiments show that the segmentation accuracy of our method does not improve significantly when we use the same batch size as other advanced convolutional neural networks.

From the experimental results of the GID dataset, we can see that our method has limited improvement compared with the Swin Transformer, and only has advantages in certain classes. When there are more computing resources, the Swin Transformer with more parameters may achieve better segmentation accuracy.

In addition, the grassland land cover type extraction model trained in this study has certain limitations. The grassland dataset we constructed in Qilian County is relatively small in size, and only a single grassland cover type is labeled. At the same time, due to the alpine shadow of the plateau and the patchy distribution of bare land and grassland, these characteristics are essentially different from grasslands in low-altitude or plain areas. Our model is only applicable to grassland land use in plateau mountainous areas. Whether our model can be directly applied to grassland extraction tasks in other low-altitude or plain areas remains to be further verified.

## Conclusions

Many existing studies aim to use remote sensing images to extract specific land cover types such as buildings (Chen et al., 2022; Dixit, Chaurasia & Kumar Mishra, 2021; Tiede et al., 2021), coastlines (Aghdami-Nia et al., 2022; Seale et al., 2022) and crop planting land (Pan et al., 2022; Zhang et al., 2021) to guide urban development, agricultural production and marine ecological conservation efforts. These studies constructed various datasets for different application areas. However, there is a lack of data to support the environmental conservation efforts for the unique terrain of the Qinghai-Tibet Plateau region. In this study, we constructed a grassland dataset for Qilian County to provide basic data support for conservation work and environmental restoration of fragile ecosystems in the plateau region.

According to the characteristics of remote sensing images, we propose a lightweight Shunted-MaskFormer for the classification of land cover types in remote sensing images. The model improves the model segmentation accuracy and reduces the model scale by using a multi-scale efficient feature extraction network and a segmentation method based on mask classification. From the experimental results, our method achieves more balanced segmentation results in different remote sensing image datasets, while obtaining optimal segmentation boundaries in the class of grassland with complex boundaries.

In this work, we basically implemented the transformer-based deep learning method for segmentation of remote sensing image land cover types. Our method uses a multi-scale global information modeling approach to reduce the model size. However, the improvement in segmentation accuracy is limited, and how to further improve the model to increase the segmentation accuracy is our next research direction in the future. In terms of dataset construction, our Qilian County grassland dataset labels the most important grassland land cover types in the study area, while the extraction of various land cover types can help us better protect the ecological environment of the Qinghai-Tibet Plateau. Therefore, we will continue to label more land cover types such as forest land and cultivated land in the future. We will also try to improve the network by using semi-supervised or unsupervised learning methods to reduce the workload of labeling.

## Additional Information and Declarations

### Data Availability

The following information was supplied regarding data availability: The Qiliangrass Data are available at Zenodo: Haiwen Chen. (2022). qiliangrassDataset (v2.0.0) [Data set]. Zenodo. https://doi.org/10.5281/zenodo.7376697. The source code is available at Zenodo: Haiwen Chen. (2022). Shunted-MaskFormer (V2.0.0). Zenodo. https://doi.org/10.5281/zenodo.7376775.
